# Neck Radiograph Halo Sign: Do Not Be Fooled

**DOI:** 10.7759/cureus.38029

**Published:** 2023-04-23

**Authors:** Nurdiana Baharudin, Hardip Gendeh, Hui Mon Teh

**Affiliations:** 1 Department of Otorhinolaryngology Head and Neck Surgery, Faculty of Medicine, Universiti Kebangsaan Malaysia, Kuala Lumpur, MYS; 2 Department of Otorhinolaryngology, Head and Neck Surgery, Hospital Queen Elizabeth, Kota Kinabalu, MYS

**Keywords:** foreign body in throat, esophageal foreign body, aerodigestive, double ring, halo sign, stacked coin, button battery

## Abstract

An aerodigestive foreign body injury in the throat is an otorhinolaryngology (ORL) emergency. Button batteries and coins are the most common foreign body aspirations or ingestions among the paediatric population. An impacted button battery in the aerodigestive tract is a surgical emergency, requiring urgent removal to prevent complications arising from the corrosive nature of the button battery. We report two cases that were brought in with a history of foreign body ingestion. Both neck radiographs showed a double-ring opaque shadow. The first child had a button battery eroding into his oesophagus. The second case is an ideally impacted stack of coins of different sizes that mimic a double-ring shadow, better known as a halo sign, in an antero-posterior neck radiograph. These cases are unique in comparing ingested coins in alignment with a button battery and a radiological examination mimicking that of a button battery. In this report, we emphasize the importance of a thorough history, endoscopic examination, and the limitations of radiographs in the initial assessment of an ingested foreign body in terms of management and prediction of morbidity.

## Introduction

Aerodigestive foreign bodies in children are often a persistent challenge to surgeons. Naturally, the curious nature of infants and young children causes them to explore objects by putting them in their mouths. In particular, an unwitnessed event often makes foreign body aspiration or ingestion a more challenging diagnosis. Quite often, an otorhinolaryngologist or emergency physician will have to depend on imaging, such as a radiograph, to predict a foreign body injury. Based on a five-year retrospective review in Romania of cases referred to a tertiary paediatric gastroenterology service, coins were the most commonly ingested foreign bodies (26%), with batteries comprising 6.6% of their cohort [1].

In young children, the smaller diameter of their oesophagus predisposes them to foreign body obstruction. More than half of all fatalities from button batteries occur after unwitnessed ingestions, reflecting a delay in detection and diagnosis. The size of a button battery might be 7.9 to 23 mm. Larger batteries (20 to 23 mm) often enter the oesophagus, while smaller button batteries pass through the stomach and intestine. However, in a very young infant with immature tracheal cartilage, dislodgement of a foreign body may compress the trachea anteriorly and result in some degree of airway compromise. Identification of the type of foreign body is important, as a button battery leads to sinister sequelae that warrant emergency removal. To confirm the diagnosis of a coin or battery in the oesophagus, anteroposterior (AP) and lateral chest radiographs are made. On the AP view, a coin within the oesophagus may align to appear as a round-shaped opacified disc. A thick vertical line from the coin's edge is observed on the lateral view [2]. Therefore, this gives rise to a "double-ring or halo sign" appearance on the AP view and a "step off" appearance on the lateral view [3].

The objectives of this manuscript are first to demonstrate a unique case whereby a radiograph may be misleading in diagnosing an ingested foreign body and, second, how a misdiagnosis may lead to the wrong assumption of emergency care and prognosis for a child with an ingested foreign body.

## Case presentation

Case 1

**Figure 1:**
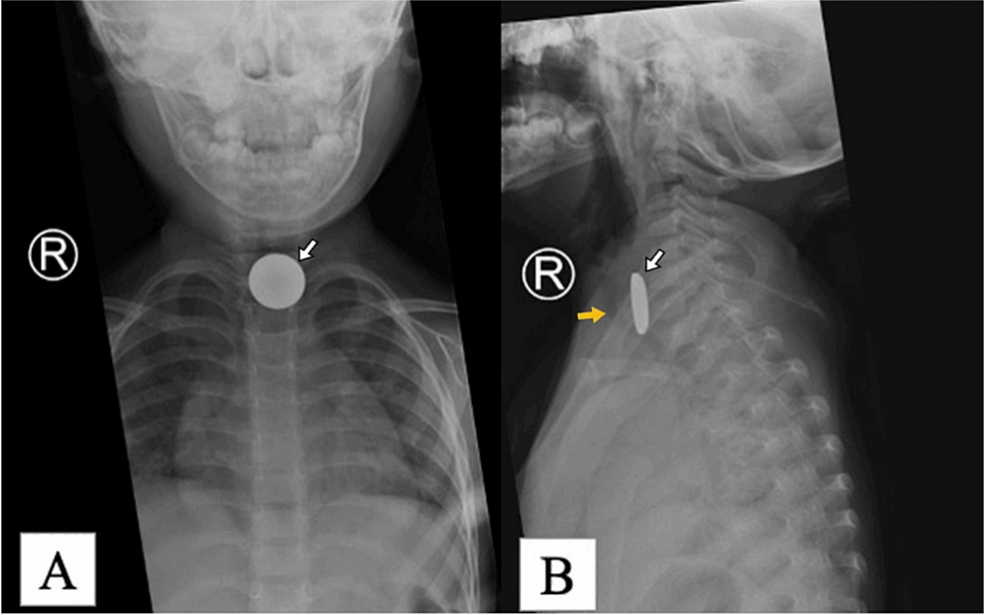
The cervical and chest X-ray (A) The anteroposterior (AP) view looks like a double ring shadow at closer view, while (B) the lateral view X-ray demonstrates the characteristic rounded edges and groove with step-off (arrow) radiopaque shadow foreign body. The view also revealed the position of foreign body in the oesophagus by located posteriorly to the trachea air shadow (yellow arrow).

**Figure 2:**
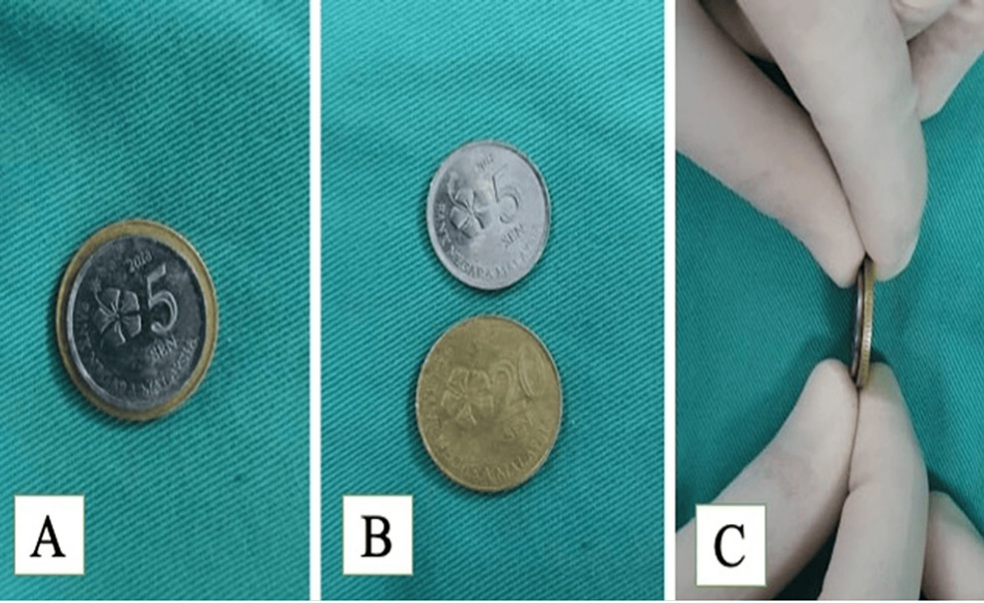
The stacked coin (A) Anterior view of the two coins stacked on each other; (B) larger coin is the twenty cent while the smaller is five cent coin; (C) lateral view of the coins stacked onto one another.

A two-year-old boy was brought to the emergency room for an acute onset of choking and salivary drooling. Parents suspected the child had swallowed a coin but were unsure of the number as they saw the coins within the vicinity. However, the child had no respiratory symptoms such as coughing, rapid breathing, or noisy breathing. A plain AP cervical and chest radiograph revealed a double-ring sign, whereby there was a double opacity in the upper third of the oesophagus (Figure 1A), highly suggestive of a button battery. On a lateral view (Figure 1B), there was an increased thickness and groove, radio-opacity, and step-off shadow. The patient underwent an emergency direct laryngoscopy, rigid oesophagoscopy, and foreign body removal within one hour of presentation. Rigid oesophagoscopy revealed a Malaysian 20-cent coin measuring 2.06 cm in diameter. Upon removal with grasping forceps, another smaller coin (a Malaysian 5-cent coin with a diameter of 1.04 cm) was discovered and retrieved. (Figure 2). A rigid oesophagoscopy was performed the second time to ensure there was no additional foreign body or trauma in the oesophagus. Postoperatively, the child was active, tolerating orally, and discharged home after one day of observation.

Case 2

**Figure 3:**
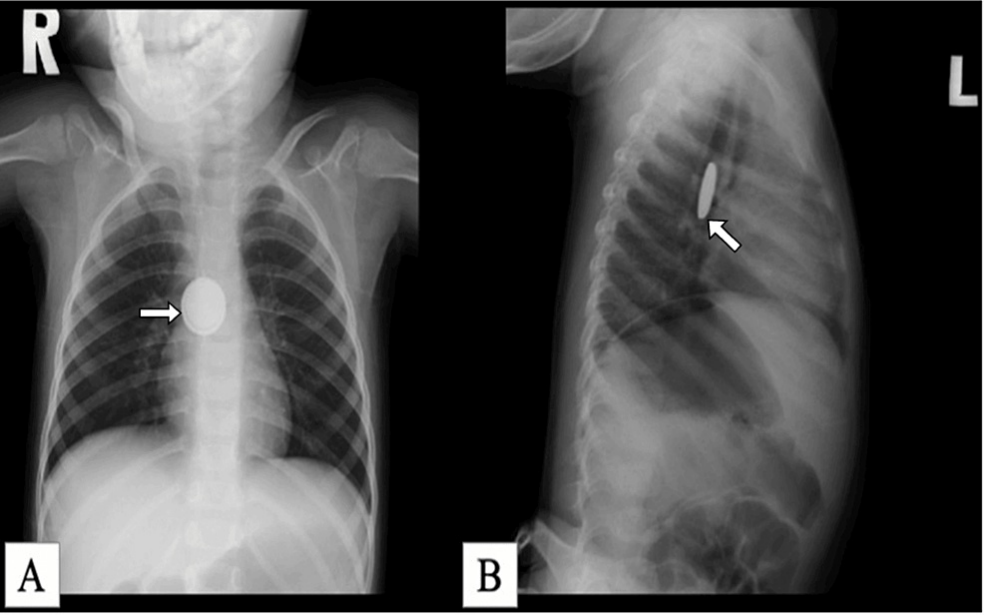
The chest and abdominal X-ray (A) The anteropesterior (AP) view revealed double ring like disc shaped radiopaque shadow in the middle third of esophagus; (B) the lateral view showed increase thickness and groove (arrow) foreign body, posterior to airway tract.

**Figure 4:**
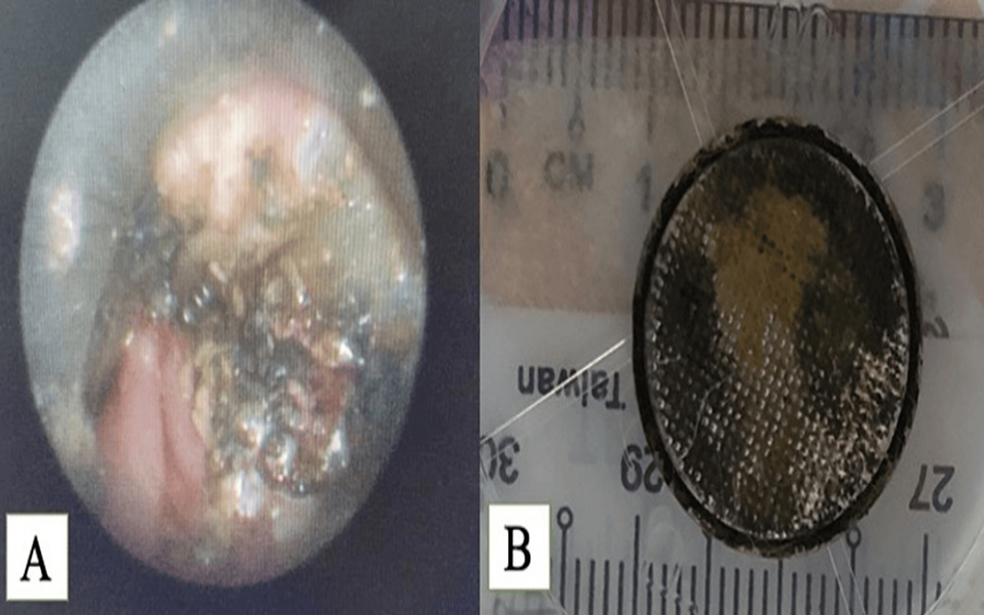
Intraoperative findings and corroded button battery retrieved from esophagus (A) Corrosion of anterior esophageal mucosa; (B) the corroded button battery of 2.0 cm diameter. Facing upward is the negative pole.

A two-year-old girl was referred for a sudden onset of dysphagia after witnessing a battery ingestion by her seven-year-old sibling. The child was comfortable until five hours later, when her parents noticed she had dysphagia with solid and semi-solid food. On arrival at the emergency department, the child was comfortable and bottle-feeding without respiratory distress. The chest and abdomen radiographs revealed the double-ring sign at the middle third of the oesophagus (Figure 3A). The lateral radiograph (Figure 3B) showed increased thickness, and a groove is appreciated within the radio-opaque shadow. Due to the geographical challenges of the mountains in Borneo, the child presented late to the hospital due to prolonged traveling times via ambulance. He underwent a direct laryngoscopy, an oesophagoscopy, and a tracheoscopy 12 hours after ingesting the foreign body. A corroded button battery of 2.0 cm in diameter was found 18 cm from the upper incisors. The button battery adhered to the anterior wall of the oesophagus, and bleeding was encountered upon removal. There was anterolateral wall erosion without fistula formation (Figure 4A). The necrotic mucosa was gently removed, and the area was irrigated with normal saline. The tracheoscopy revealed no erosion, cartilage exposure, or fistula over the tracheal mucosa. A nasogastric tube was inserted, and the child was kept fasted for five days with a prescription of intravenous co-amoxiclav and proton pump inhibitors. Barium swallowing was normal prior to the removal of the nasogastric tube. The child was discharged well and did not suffer complications.

## Discussion

**Figure 5:**
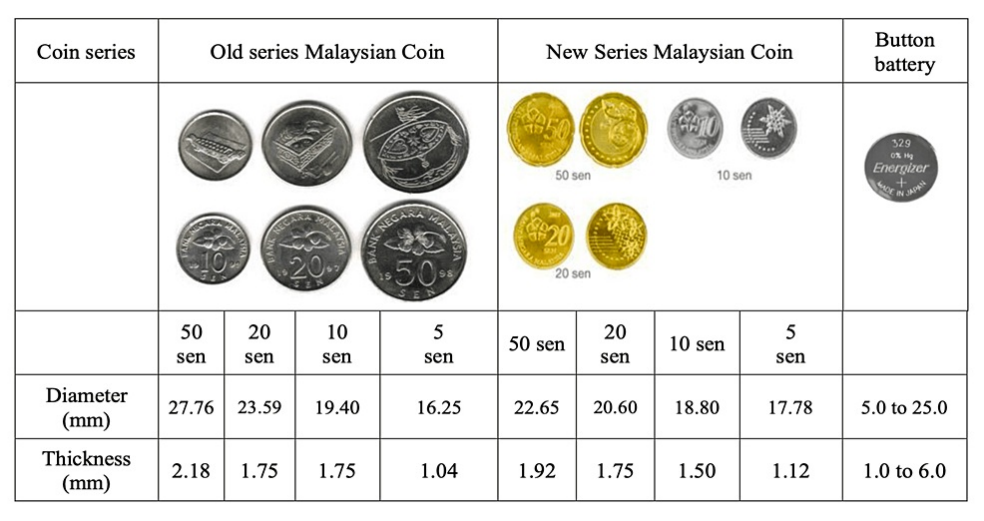
Comparison size between Malaysian coin series and button battery

To date, button or coin batteries have become one of the essential power sources for many objects at home, including watches, calculators, hearing aids, and electronic toys. The battery comes in various sizes, but most are 1 to 2.5 cm in diameter and 1 to 6 mm thick [4,5]. Despite moving towards a modern digital economy, coins remain relevant, especially among rural communities or low-income people. This community finds cash easier to manage because of poor broadband and mobile connectivity. Thus, coins become relevant and easily accessible. The table in Figure 5 below illustrates the diameters and thicknesses of Malaysian coins, which come in the denominations of 5 sen, 10 sen, 20 sen, and 50 sen. The size of each coin proportionately increases with its value and has a diameter of 1.7 to 2.5 cm.

The glossy features of coins and button batteries and their resemblance to the size of candy or sweets can be appealing for tasting and ingestion. The majority of foreign body ingestion is expected in children aged two to five years old due to mouthing behaviour.

Both button batteries and coins are made of metal, which gives the radiopaque circle shadow in the radiograph (AP view) its appearance. In an anteroposterior (AP) radiograph, the button battery and coin are presented as round-shaped radiopaque shadows. However, a double rim or halo sign is seen in the button battery compared to the homogenous shadow in the coin. Though the double rim is not only exclusive to the button battery, the sign can also be simulated by a pot magnet and an ideally impacted double coin, as presented in this case [5]. Both the AP and lateral radiographs for batteries in the oesophagus are critical to determining the orientation of the positive and negative poles. The double ring shadow is presented by the inner shadow of the negative pole with a slightly smaller diameter, fitting within the battery can and forming the outer ring shadow from the positive pole [6]. The double-rim appearance has been unique to a button battery and, to date, has been a reliable predictor of a button battery. This is the first case whereby the halo sign was misleading and a poor predictor of a button battery; instead, it reveals a perfectly superimposed pair of coins of different diameters located side by side.

To aid in the diagnosis, a lateral soft tissue neck X-ray can confirm the position of the foreign body in the oesophagus, posterior to the trachea, and the step-off shadow, which is on the negative pole of the battery [6], helps to differentiate it from a coin. However, when different sizes of coins are stacked side by side, a step-off shadow is formed, thus mimicking that of the button battery [3,6]. The ability to differentiate foreign bodies is vital, as, mainly in Malaysia, the radiograph interpretation is made chiefly by the clinician, not requiring a radiologist's report, more so for a remote and smaller area within Borneo.

A button battery initially presents similarly to coin ingestion. Given the risk of severe electrochemical burns within two hours of post-ingestion of a button battery, the current standard of care for treating batteries in the oesophagus is emergent endoscopic removal, regardless of whether the patient is symptomatic [7,8].

Batteries may cause local trauma via three mechanisms. First, pressure necrosis - direct pressure on the mucous membrane; second, chemical damage due to leakage of corrosive battery contents (often alkaline) and electrical damage from small electrical circuits (eddy currents) generated by contact of the battery poles against the moist mucus membrane in the oesophagus [8]. The eddy current of the button battery, conducted through saliva and the tissue, drives a highly alkaline caustic injury, leading to liquefaction, tissue necrosis, and subsequently perforation.

In the second case, removing the button battery was delayed until 12 hours post-ingestion. The delay in presenting to the hospital is due to parents' lack of understanding about the harm caused by button battery consumption. A logistical issue resulted from delayed ambulance availability in the Sabah, Borneo, Malaysia district hospitals and a long journey from the rural district hospital to the tertiary, which took at least two hours. While awaiting retrieval, the child was kept nil by mouth with intravenous fluid hydration.

Nevertheless, removing the button battery should not be delayed in an emergency setting, even if the child was not kept nil by mouth. Administration of honey or sucralfate orally is proposed to help neutralize the tissue before removal and effectively reduce the damage to the tissue. In order to mitigate the severity of the oesophageal burn, irrigation with 50-150 ml of 0.25% sterile acetic acid solution should be used intraoperatively to neutralize the tissue [3,8].

A nasogastric tube feeding post-button battery removal is essential to reduce complications such as oesophageal-tracheal fistulae that may occur several days after removal and oesophageal stenosis due to scarring that may occur weeks to months after ingestion. Empirical antibiotic therapy is administered to prevent secondary infections. A review of the literature reveals that there is still no conclusive proof to support the use of prophylactic antibiotics such as amoxicillin/clavulanate (Augmentin) or ampicillin/sulbactam (Unasyn) in patients who ingested a button battery. However, in such circumstances, the antibiotics prescribed should cover both the normal oropharyngeal flora and *Staphylococcus aureus* [9].

The management of post-button battery removal has ripened for debate. Although some have cited the value of a second-look endoscopy two to four days post-operatively to determine the timing of feeding introduction, such timing for a second look may lead to false reassurance about continued risks for complications. The risk of complications can happen as the perforations are usually diagnosed within 48 hours and rarely in the first 12 hours, but fistulas can present up to four weeks post-removal [9]. Here, rigid oesophagoscopy and tracheoscopy were performed in the first operation, followed by the upper GI contrast study on day five post-removal, which revealed no fistula or stricture. This procedure is an excellent non-invasive investigation that can be used to evaluate the healing process of the oesophagus. In addition, this also reduces the child's exposure to anesthesia for repeated oesophagoscopy. The patient must watch out for any delayed sequelae for a proper follow-up.

In addressing the risk associated with button batteries, legislation, and industry changes are crucial to limiting the risks of button battery ingestion among children and the sinister complications that may follow. In the line to prevent and minimize button battery injury, the National Button Battery Taskforce and some industry members have introduced the strategies such as educational safety campaigns, child-resistant packaging, and warning labels [10]. Safety standards now regulate locked battery compartments in toys and, with technological advancement, a coated battery. The legislation should be made it compulsory for industries to provide a coated battery, making it less appealing and unappetizing. The current available coated battery, such as quantum tunneling composite coated (QTCC), has been shown to minimize the risk of electrochemical burn once ingested. These coated batteries are nonconductive in the low-pressure gastrointestinal environment but conduct in ordinary battery housings. Therefore, the coated battery should be made widely available as the most significant and cost-effective; this coated battery can be used in any battery-operated gadget [11].

## Conclusions

The double ring appearance or halo sign in the radiograph of a child with a history of foreign body ingestion may not always be a button battery. However, it can be a stack of coins with different diameters. It is fundamental to get an accurate and thorough history with an examination, aided by a comparative evaluation of radiographs. It gives clues to the diagnosis and management.
